# Increased myocardial dysfunction, dyssynchrony, and epicardial fat across the lifespan in healthy males

**DOI:** 10.1186/1471-2261-14-95

**Published:** 2014-08-03

**Authors:** Edward Crendal, Fred Dutheil, Geraldine Naughton, Tracey McDonald, Philippe Obert

**Affiliations:** 1 School of Exercise Science, Australian Catholic University, Locked Bag 4115, Fitzroy, MDC Victoria 3065, Australia; 2 Laboratory of Pharm-Ecology Cardiovascular EA4278, School of Sport Sciences and Exercise, University of Avignon, Avignon, France; 3 Laboratory of Metabolic Adaptations to Exercise in Physiological and Pathological conditions EA3533, Blaise Pascal University, Clermont-Ferrand, France; 4 Occupational Medicine, University Hospital CHU G. Montpied, Clermont-Ferrand, France

**Keywords:** Aging, Speckle tracking echocardiography, Dyssynchrony, Myocardial dysfunction, Epicardial fat

## Abstract

**Background:**

Evaluation of sensitive myocardial mechanics with speckle tracking echocardiography (STE) across the lifespan may reveal early indicators of cardiovascular disease (CVD) risk. Epicardial adipose tissue (EAT) and left ventricular (LV) myocardial dyssynchrony; subclinical risk-factors of CVD, are of particular clinical interest. However, the evolution of EAT and LV-dyssynchrony across the lifespan, and their influence on myocardial dysfunction remains unclear. We aimed to establish a profile of the healthy aging-heart using conventional, tissue-Doppler imaging (TDI) and speckle-tracking echocardiography (STE), while also exploring underlying contributions from EAT and LV-dyssynchrony towards LV myocardial mechanics, independent of blood biology.

**Methods:**

Healthy males aged 19–94 years were recruited through University-wide advertisements in Victoria and New-South Wales, Australia. Following strict exclusion criteria, basic clinical and comprehensive echocardiographic profiles (conventional, TDI and STE) were established. LV-dyssynchrony was calculated from the maximum-delay of time-to-peak velocity/strain in the four LV-annulus sites (TDI), and six LV-segments (STE longitudinal and circumferential axes). Epicardial fat diameter was obtained from two-dimensional grey-scale images in the parasternal long-axis. Blood biological measures included glycemia, hsCRP, triglycerides, total cholesterol, high-density and low-density lipoprotein levels.

**Results:**

Three groups of 15 were assigned to young (<40 years), middle (40–65 years), and older (>65) aged categories. Five participants were excluded from STE analyses due to inadequate image quality. Decreased longitudinal strain, increased circumferential apical strain and LV twist were age-related. Moreover, independent of blood biology, significant increases were observed across age categories for EAT (young: 2.5 ± 0.9 mm, middle: 3.9 ± 1.0 mm, older 5.7 ± 2.4 mm; p < 0.01), longitudinal STE-dyssynchrony (young: 42 ± 7.7 ms, middle: 58.8 ± 18.9 ms, older 88.6 ± 18.2 ms; p < 0.05), and circumferential-basal STE-dyssynchrony (young: 50.2 ± 20.5 ms, middle: 75.9 ± 20.6 ms, older 97.9 ± 20.2 ms; p < 0.05). These variables collectively explained 37% and 31% (p < 0.01) of longitudinal strain and LV twist, respectively.

**Conclusions:**

This study enabled comprehensive profiling of LV mechanics at different stages of aging using sensitive echocardiographic technology. Novel findings included increased epicardial fat, and both longitudinal and circumferential LV-dyssynchrony across the healthy age groups. These factors may be key underlying contributors to myocardial dysfunction during aging, and their recognition may promote an advanced understanding of early signs of cardiovascular disease.

## Background

Increased life expectancy and decreased birth rates contribute to the notion of global population aging [1,2], with cardiovascular disease (CVD) remaining the primary contributor to mortality [3]. A better understanding of the healthy aging-heart may support the early prevention, and thus reduced burden, of CVD. Evidence suggests that normal aging is associated with left ventricular (LV) hypertrophy and decreased diastolic LV function, assessed with conventional and tissue Doppler imaging (TDI) echocardiography [4-6]. The use of speckle tracking echocardiography (STE) may permit more sensitive analyses of the aging-heart, through the angle-independent assessment of myocardial deformation (myocardial mechanics) in the longitudinal and circumferential axes, as well as twist mechanics [7]. Previous studies of LV myocardial mechanics across the lifespan report an age-related decrease in longitudinal deformation, as well as compensatory increases in LV twist using STE [8-13]. Meanwhile, the evolution of *circumferential deformation* with age remains inconclusive.

Underlying contributors to these LV changes in healthy aging populations are poorly understood. Specifically, epicardial adipose tissue (EAT), measured feasibly by echocardiography, is strongly associated with myocardial lipid content; a potentially major contributor to LV dysfunction in populations with metabolic disorders [14,15]. However, the influence of EAT on LV myocardial mechanics in healthy older people is unknown and warrants further attention since it may provide an early indication of CVD risk.

Furthermore, non-uniform contraction of LV myocardial walls due to declining electromechanical synchronicity (LV-dyssynchrony) may result in myocardial inefficiency [16], and could also be an underlying contributor to these age-related impairments in LV myocardial mechanics. Previous studies have found increasing circumferential [17], longitudinal and radial [18] LV-dyssynchrony with age. Nonetheless, no studies have examined the association of both longitudinal and circumferential LV-dyssynchrony with STE-derived indices of myocardial mechanics, such as LV longitudinal and circumferential strain as well as twist, in a healthy aging population.

The primary aim of this cross-sectional study was therefore to establish a comprehensive profile of LV mechanics across different age categories of healthy males, using conventional, TDI and STE echocardiography. A secondary aim was to explore how EAT and LV-dyssynchrony change with age, and quantify their underlying contribution to important measures of LV myocardial mechanics, independently of blood-biology, within this population.

## Methods

### Population and design

Participants provided informed consent prior to commencement of testing, and the study protocol conformed to the ethical guidelines of the 1975 Declaration of Helsinki as reflected in a prior approval by the Human Research Ethics Committee (HREC) of the Australian Catholic University, Melbourne, Australia. Volunteers were recruited through advertisements, and all participants provided informed consent. Inclusion criteria were males aged over 18 years with no maximum age limit, no prior cardiovascular disease history, diabetes mellitus, dyslipidemia, or hypertension, no routine medication, and (to avoid highly trained athletes) practising less than 10 hours per week of moderate to intense physical activity. Volunteers were screened with verbal interviews and written questionnaires. We recruited males from three age categories: younger than 40 years (Y); 40 to 65 years (M); and older than 65 years (O).

### Clinical and biochemical parameters

Stature, body mass, and body mass index (BMI) were recorded. Blood pressure was measured with a digital sphygmomanometer (Carescape-V100, Dinamap, General Electric technology, USA) after 15 minutes lying in the supine position. Physical activity was quantified using the International Physical Activity Questionnaire modified for elderly populations [19].

Fasted serum concentrations of glucose, triglycerides, total cholesterol, high-density lipoprotein (HDL), low-density lipoproteins (LDL), and high-sensitivity C-reactive protein (hsCRP) were analysed at a clinical pathology laboratory (Melbourne Pathology, Australia).

### Conventional echocardiography

Participants underwent transthoracic echocardiogram and electrocardiogram examination by the same experienced operator, following standard recommendations [20]. Images and cine-loops were obtained using an equipment pack (Vivid-i, General Electric, Milwaukee, WI, USA) and a 3.5-MHz probe. Images were digitally recorded for subsequent offline analysis with dedicated software (EchoPAC PC, Version 5, General Electric Healthcare). Each variable was measured on an average of three consecutive cardiac cycles. M-Mode measurements were obtained in the parasternal long-axis view. Left atrial (LA) and LV dimensions were measured at end-diastole and end-systole. LV mass was calculated by the Devereux formula, and indexed for height [21], and LV ejection fraction was derived from semiautomatic quantification of LV volumes, using STE. Pulsed-Doppler LV transmitral blood velocity, including early (E) and atrial (A) waves, were measured in the apical four-chamber view. Isovolumic relaxation time was measured by pulsed Doppler in the apical five-chamber view, along with aortic ejection velocity. Spectral pulsed-TDI measures of myocardial systolic (S_m_), early diastolic (E_m_), and late diastolic (A_m_) velocities were assessed at the mitral annulus of the LV, in apical four (septal and lateral) and two (anterior and inferior) chamber views. The E/E_m_ ratio, recorded from the mitral annulus lateral wall, was used as an index of LV filling pressure. The tricuspid annular plane systolic excursion (TAPSE) and TDI S_m_ velocity were also assessed on the lateral (free) wall of the right ventricle. EAT diameter was manually measured on the free wall of the right ventricle from the two-dimensional grey-scale image in the parasternal long-axis view, as previously described [22].

### Speckle tracking echocardiography

The STE mode of two-dimensional strain imaging was used to measure more sensitive myocardial mechanics. Offline analyses were performed in the circumferential axis at the base, papillary-muscle level, and apex of the LV (parasternal short-axis), as well as in the longitudinal axis (apical four-chamber view), using software described above. In the respective B-mode two-dimensional grey-scale cine-loops, a region of interest was manually placed along the endocardial border, and automatically covered myocardial thickness to the epicardial border. Six segments were subsequently analysed in each view. Cine-loops without adequate endocardial border definition, frame rate (>60 Hz), and image tracking were excluded from analyses. The cine-loops were animated, and LV longitudinal strain, strain rate (SR), and time to peak strain/SR were obtained in the apical four-chamber view. Circumferential strain, SR, time to peak strain/SR, and rotation were obtained from the short-axis view at the LV base and apex. LV twist was calculated as the instantaneous difference between apical and basal rotation. To adjust all STE variables for inter-participant differences in heart rate, the time sequence was normalized to the percentage of systolic duration (i.e. 100% at aortic valve closure) using a toolbox generated in our laboratory (Scilab 4.1) [23]. Inter and intra-observer reproducibility were estimated in three views from 10 randomly selected participants, and the coefficient of variations were 5.4% and 5.1%, respectively for strain, and 10.1% and 7.9%, respectively for twist.

### Left ventricular dyssynchrony

TDI LV-dyssynchrony was measured by recording time taken from the start of QRS complex to peak S_m_ (systolic dyssynchrony) or peak E_m_ (diastolic dyssynchrony) at the annulus of the septal and lateral walls (apical four-chamber view), and inferior and anterior walls (apical two-chamber view). For STE LV-dyssynchrony, time taken from the start of QRS to peak strain in the six LV segments was measured in both longitudinal (apical 4-chamber) and circumferential (parasternal short-axis basal and apical levels) axes. LV-dyssynchrony was evaluated using the maximum delay technique in the four (TDI) or six (STE) segments. This modality of measuring LV-dyssynchrony is more sensitive than the Yu index or opposing wall delay [24]. Clinical diagnosis was made if maximum delay was greater than 65 ms [25] and 100 ms [26] for TDI and STE, respectively. For STE dyssynchrony, inter and intra-observer reproducibility (coefficient of variation) were 9.3% and 10.2%, respectively.

### Statistical analyses

It was predicted that a minimum of 13 participants in each population sub-group (Y, M, and O) would allow detection of a significant difference if the mean of the major cardiac variables was between 1 and 1.25 standard deviations higher than the mean value between the groups (80% power; p < 0.05) [26]. After verifying Gaussian distribution, clinical, biological, and echocardiographic data were presented as means (standard deviation). One-way analysis of variance (ANOVA) with Tukey’s post-hoc tests were used to assess differences between Y, M, and O groups. In order to preclude outliers, we excluded data with values beyond two standard deviations of the group mean [27]. Pearson’s *r* correlation analyses were performed between age, cardiac variables, and blood biology. Univariate analysis of co-variance (ANCOVA) was performed to test the strength of the relationships between LV-dyssynchrony and age; after the effects of glucose, total cholesterol and hsCRP were controlled. A stepwise linear regression model assessed the predictive role of LV-dyssynchrony on major indices of myocardial mechanics (LV twist and longitudinal strain). Analyses were performed using SPSS Version 16.0 for windows (SPSS Inc), with statistical significance set at p < 0.05.

## Results

### Participants

**Figure 1:**
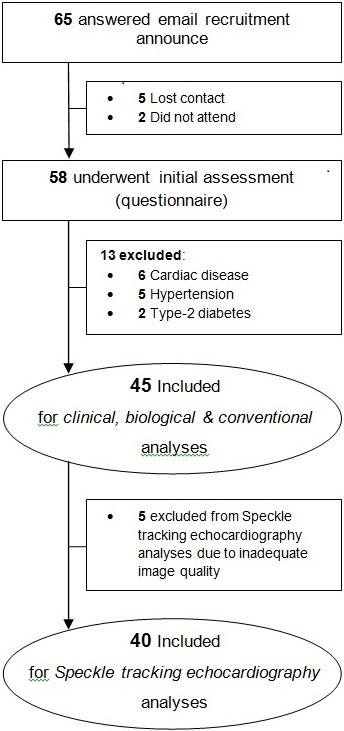
Participant recruitment flow chart.

Participant recruitment is described in Figure 1. Forty-five healthy males took part in the study. Fifteen participants per group were assessed using conventional, TDI, and longitudinal STE echocardiography. One (Y), two (M) and two (O) participants were excluded for circumferential STE analysis due to insufficient image quality. Normal sinus rhythm, with no widening of QRS morphology was present in all participants.

### Clinical and biochemical parameters

Mean ages of participants were 25.7 ± 6.1 (Y), 49.3 ± 8.8 (M) and 77.7 ± 9.8 (O). Participant age ranged from 19 to 94 years. Age groups did not differ in body mass, BMI, systolic and diastolic blood pressure, hsCRP, triglycerides, HDL, LDL, total cholesterol, and glucose. Stature was lower in O than M and Y groups (p < 0.05) (Table 1).

**Table 1:** Clinical and biological parameters of healthy males

	**Young**	**Middle**	**Older**
	**< 40 years old**	**40-65 years old**	**> 65 years old**
	**(n = 15)**	**(n = 15)**	**(n = 15)**
Age (years)	25.7 (6.1)	49.3 (8.8)***	77.7 (9.8)***###
Stature (cm)	180.5 (5.4)	177.9 (4.9)	172.6 (2.3)*#
Body mass (kg)	80.2 (8.9)	83.1 (11.7)	76.7 (9.3)
BMI (kg.m^-2^)	24.6 (3.0)	25.9 (3.5)	25.7 (3.0)
Systolic BP (mmHg)	124.5 (8.3)	125.1 (9.8)	122.4 (11.4)
Diastolic BP (mmHg)	73.6 (8.5)	79.6 (6.9)	73.1 (9.0)
Fasting glucose (mmol.L^-1^)	5.1 (0.3)	5.3 (0.4)	5.2 (1.0)
HDL (mmol.L^-1^)	1.4 (0.2)	1.5 (0.5)	1.3 (0.2)
LDL (mmol.L^-1^)	2.6 (1.1)	3.0 (0.9)	3.1 (0.9)
Total cholesterol (mmol.L^-1^)	4.4 (1.2)	4.9 (1.0)	5.1 (1.0)
Triglycerides (mmol.L^-1^)	0.8 (0.4)	1.0 (0.6)	1.3 (0.6)
High sensitivity CRP (mg.L^-1^)	0.7 (0.4)	1.0 (0.7)	1.8 (1.7)

Data are means (standard deviations). BMI: Body mass index, BP: Blood pressure, HDL: High density lipoprotein, LDL: Low density lipoprotein, CRP: C-reactive protein. *: p < 0.05 vs. Young; ***: p < 0.001 vs. Young; #: p < 0.05 vs. Middle; ###: p < 0.001 vs. Middle.

### Conventional echocardiographic and TDI parameters

Results from *LV remodeling* (Table 2) showed only interventricular septum thickness and relative wall thickness (RWT) were greater in O than Y (p < 0.05), but neither differed from M. RWT was also greater in M than Y (p < 0.05).

**Table 2:** Conventional and tissue Doppler imaging echocardiography parameters of healthy males

	**Young**	**Middle**	**Older**
	**< 40 years old**	**40-65 years old**	**> 65 years old**
	**(n = 15)**	**(n = 15)**	**(n = 15)**
LV M-Mode			
HR (beats.min-1)	56.8 (8.1)	59.1 (6.0)	60.6 (14.1)
LAD diameter (mm)	35.7 (4.6)	35.6 (3.9)	36.3 (5.5)
LVED diameter (mm)	52.9 (3.7)	50.0 (3.5)	50.3 (3.5)
LVES diameter (mm)	34.9 (4.0)	32.1 (5.3)	33.7 (5.6)
IVSD diameter (mm)	10.5 (0.5)	11.5 (1.4)	11.8 (1.2)*
PWD diameter (mm)	10.6 (0.9)	11.3 (1.3)	11.1 (1.1)
RWT (mm)	0.40 (0.1)	0.45 (0.1)*	0.46 (0.1)*
LV mass (g)	221.6 (36.3)	216.5 (38.4)	229.4 (42.5)
LV mass index (g.m^-2.7^)	44.9 (6.4)	45.7 (7.7)	52.9 (9.3)*#
LV ejection fraction (%)	63.5 (5.2)	64.2 (6.5)	63.1 (6.9)
LV Pulsed-Doppler			
E velocity (cm.s^-1^)	75.1 (13.1)	67.5 (9.3)	62.6 (14.7)*
A velocity (cm.s^-1^)	40.4 (6.6)	55.9 (11.7)*	66.8 (25.8)***
E/A	1.9 (0.5)	1.3 (0.4)***	1.0 (0.3)***
E deceleration time (ms)	163.9 (22.4)	168.1 (16.5)	191.9 (38.8)*
IVRT (ms)	94.3 (11.7)	110.8 (16.4)*	115.6 (25.0)*
LV Pulsed-TDI			
E_m_ (cm.s^-1^)	14.2 (2.2)	10.3 (2.8)***	8.4 (2.2)***
A_m_ (cm.s^-1^)	6.6 (0.8)	8.0 (1.7)	9.0 (2.4)*
S_m_ (cm.s^-1^)	8.0 (0.7)	7.4 (1.5)	7.5 (1.3)
E/E_m_	4.9 (0.8)	6.1 (1.7)	7.4 (2.7)*
Right ventricle			
EAT (mm)	0.25 (0.09)	0.39 (0.10)*	0.57 (0.24)***#
RV S_m_ (cm.s^-1^)	13.6 (1.7)	11.2 (1.5)*	11.7 (2.2)*
TAPSE (mm)	22.8 (1.8)	20.6 (1.4)	21.6 (3.4)

Data are means (standard deviations). LV: Left ventricular, HR: Heart rate, LAD: Left atrial diastolic, LVED: Left ventricular end diastolic, LVES: Left ventricular end systolic, IVSD: Interventricular septum diastolic, PWD: Posterior wall diastolic, RWT: Relative wall thickness, IVRT: Isovolumic relaxation time, EAT: Epicardial adipose tissue, RV: Right ventricle, TAPSE: Tricuspid annular plane systolic excursion. *: p < 0.05 vs. Young; ***: p < 0.001 vs. Young; #: p < 0.05 vs. Middle; ###: p < 0.001 vs. Middle.

Among results from *functional LV parameters*, no group differences were observed in systolic function (LV ejection fraction and S_m_). However, diastolic function was impaired across the age groups. E/A ratio was lower in O than both M and Y (p < 0.001), without differences between M and Y. E_m_ was lower in both O and M than Y (p < 0.001), without differences between O and M. Deceleration time of E and LV filling pressure (E/E_m_) were greater in O than Y (p < 0.05), but not different from M. Isovolumic relaxation time was more prolonged in both O and M than Y (p < 0.05), but O and M were similar.

Results from the *RV analyses* (Table 2) showed no inter-group differences for tricuspid annular plane systolic excursion TAPSE. However, S_m_ was lower in both O and M than Y (p < 0.05), without differences between O and M. EAT diameter was greater in O than both M and Y (p < 0.001), while M was also greater than Y (p < 0.001).

### Speckle tracking echocardiographic parameters

For *longitudinal* strain and diastolic SR (Table 3), O was lower than M and Y, respectively (p < 0.001 and p < 0.05), while no differences were observed between M and Y. Longitudinal systolic SR was unaltered by age. Despite no between group differences for basal *circumferential* characteristics; apical circumferential strain and systolic SR were greater in O and M than Y (p < 0.05). However, apical circumferential strain was similar in O and M. Apical circumferential diastolic SR was unaltered by age (Table 3). Further analyses of circumferential mechanics showed no differences across the lifespan for basal rotation. However, apical rotation was greater in O and M than Y (p < 0.05). Subsequent LV twist and untwist rates were greater in O than Y (p < 0.05), but not different from M.

**Table 3:** Left ventricular speckle tracking echocardiography parameters of healthy males

	**Young**	**Middle**	**Older**
	**< 40 years old**	**40-65 years old**	**> 65 years old**
	**(n = 14)**	**(n = 13)**	**(n = 13)**
Longitudinal axis			
Strain (%)	-20.6 (2.4)	-19.3 (2.9)	-15.7 (3.4)***###
Diastolic SR (%.sec^-1^)	1.5 (0.3)	1.3 (0.3)	1.1 (0.2)***#
Systolic SR (%.sec^-1^)	-1.0 (0.2)	-1.0 (0.2)	-0.9 (0.2)
Base circumferential			
Strain (%)	-16.5 (3.7)	-16.8 (5.9)	-15.8 (2.6)
Diastolic SR (%.sec^-1^)	1.5 (0.3)	1.6 (0.4)	1.2 (0.3)
Systolic SR (%.sec^-1^)	-1.0 (0.2)	-1.1 (0.3)	-1.1 (0.3)
Apex circumferential			
Strain (%)	-21.4 (2.7)	-26.0 (5.8)*	-26.8 (3.0)*
Diastolic SR (%.sec^-1^)	1.8 (0.3)	2.2 (0.9)	2.0 (0.4)
Systolic SR (%.sec^-1^)	-1.2 (0.2)	-1.5 (0.3)*	-1.7 (0.3)*
Rotational mechanics			
Apical rotation (°)	4.1 (2.1)	6.4 (2.2)*	6.1 (2.1)*
Basal rotation (°)	-4.6 (1.6)	-5.5 (2.5)	-6.4 (3.1)
Peak twist (°)	6.6 (2.2)	8.1 (3.6)	11.6 (2.1)*
Peak untwist-rate (°.sec^-1^)	-65.0 (19.5)	-82.9 (16.0)	-92.4 (22.1)*

Data are means (standard deviations). SR: Strain rate. *: p < 0.05 vs. Young; ***: p < 0.001 vs. Young; #: p < 0.05 vs. Middle; ###: p < 0.001 vs. Middle.

### Left ventricular dyssynchrony parameters

**Figure 2:**
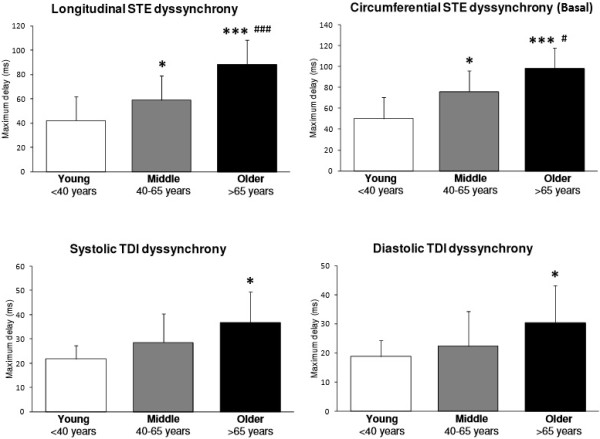
**Data are means ± standard deviations.** Left ventricular dyssynchrony derived from STE or TDI across the lifespan in healthy males. STE: Speckle tracking echocardiography. TDI: Tissue Doppler imaging. L: Longitudinal. C: Circumferential (basal level). *: p < 0.05 vs. Young; ***: p < 0.001 vs. Young; #: p < 0.05 vs. Middle; ###: p < 0.001 vs. Middle.

Measures of LV-dyssynchrony across the age groups are presented in Figure 2. Both *TDI-derived* systolic and diastolic maximum delay were greater in O than Y (p < 0.05). TDI-derived maximum delay did not differ between O and M, nor M and Y. In contrast, *STE-derived longitudinal* maximum delay was greater in O than both M and Y (p < 0.001), while also greater in M than Y (p < 0.05). Additionally, *STE-derived circumferential* maximum delay at the base was greater in O than both M and Y (p < 0.05), while also greater in M than Y (p < 0.05). No inter-group differences existed for STE-derived circumferential maximum delay at the apex.

### Relationships amongst cardiac variables, age, and blood biology

Age was significantly associated with the following cardiac variables: longitudinal STE LV-dyssynchrony (*r =* 0.79), TDI E_m_ (*r =* 0.78), circumferential-basal STE LV-dyssynchrony (*r =* 0.77), E/A ratio (*r =* 0.75), EAT (*r =* 0.73), LV twist (r = 0.68), and longitudinal strain (*r =* 0.62) (all p < 0.01). Age was also significantly related to hsCRP (*r =* 0.40) and triglycerides (*r =* 0.35) (both p < 0.05). Furthermore, some cardiac variables also correlated strongly with blood biology, including; apical circumferential rotation and TDI A_m_ with total cholesterol (*r =* 0.47 and *r =* 0.46, respectively), apical circumferential systolic SR with hsCRP (*r =* 0.42), TDI E_m_ with fasting glucose (*r =* 0.42), and EAT with triglycerides (*r =* 0.42) and hsCRP (*r =* 0.40) (all p < 0.05). After controlling for total cholesterol, fasting glucose, and hsCRP; age remained correlated to longitudinal STE LV-dyssynchrony (*adjusted r =* 0.72; p < 0.001).

### Relationships between LV-dyssynchrony, EAT and indices of myocardial mechanics

Longitudinal LV-dyssynchrony was associated with longitudinal strain (*r =* 0.51; p < 0.01) while circumferential STE LV-dyssynchrony at the base was associated with LV twist (*r =* 0.52 p < 0.05). Circumferential STE LV-dyssynchrony at the apex was not associated with any indices of myocardial mechanics. EAT was also associated with longitudinal STE LV-dyssynchrony (*r =* 0.65), TDI E_m_ (*r =* 0.60), longitudinal strain (*r =* 0.50), circumferential STE LV-dyssynchrony at the base (*r =* 0.44), and LV twist (*r =* 0.44) (all p < 0.01). A stepwise linear regression model revealed that the combination of EAT, longitudinal STE LV-dyssynchrony, and circumferential STE LV-dyssynchrony at the base explained 37% of the variability in longitudinal strain, and 31% of the variability in LV twist (*R*^*2*^*=* 0.37 and 0.32, respectively; p < 0.05).

## Discussion

We investigated cardiac profiles in healthy males of increasing age, using conventional and STE imaging technologies. This enabled several major findings to emerge. First, we confirmed a decline in global diastolic function and longitudinal deformation, accompanied by compensatory increases in circumferential apical strain and LV twist across the three age-groups. Additionally, for the first time with echocardiography, we showed increasing EAT, as well as longitudinal and circumferential-basal LV-dyssynchrony across the three age-groups, independent of the effects of blood biology. Moreover, these three factors emerged as significant correlates and predictors of key myocardial mechanics parameters (longitudinal strain and LV twist).

### Rationale for elderly male population

Previous studies of LV myocardial function and dyssynchrony in aging people have not observed individuals older than 89 years of age [8,9,11-13,17,18,28]. In the present study, our participants were aged up to 94 years. We demonstrated a linear increase in LV myocardial dysfunction and dyssynchrony across the three incremental age groups. Furthermore, prior studies of the aging-heart observed mixed gender populations. We present for the first time, a study of males only. Evidence suggests myocardial tissue in females is more preserved than in males throughout aging [29]. We demonstrated that restriction to a male-only population minimised standard deviations and permitted detection of significant differences between groups, with fewer individuals and with an analysis free from hormonal confounders found in mixed gender populations [28].

### Aging and conventional echocardiographic parameters

The present study found mild LV hypertrophy and more impaired diastolic function in the O group than M and Y groups, supporting other longitudinal studies showing LV remodeling and diastolic dysfunction across the lifespan, despite preserved LV ejection fraction [4-6,30]. The fundamental mechanisms behind these changes may be due to age-associated increases in arterial blood pressure and subsequently elevated afterload [31]. However, although E/E_m_ ratio (a measure of LV filling pressure) was elevated in O participants, both systolic and diastolic arterial blood pressures were similar across the age-groups. Analysis of cardiac function at the more regional (myocardial) level was therefore justified.

### Aging and myocardial mechanics

We revealed decreasing TDI-derived diastolic myocardial velocity across the age-groups, agreeing with other aging studies [32-35]. We also confirmed previous findings of preserved TDI systolic velocity across the age-groups [32,36,37], despite some conjecture in the literature [33,38,39]. The exclusion of hypertensive individuals in the current but not previous studies may explain this discrepancy since blood pressure strongly influences systolic function [40]. Furthermore, longitudinal deformation (strain and both systolic and diastolic SR) was impaired in older healthy males, supporting previous studies [11,41,42]. Most likely in response to the impaired longitudinal deformation, we observed compensatory increases in apical circumferential deformation and LV twist with age. This would help explain the observed preservation of LV ejection fraction [11,12,36]. Our findings agree with recent evidence establishing age-related normal values in longitudinal strain and LV torsion using three-dimensional STE [13]. The authors found an age-related decline in longitudinal function, and increased LV torsion, concluding that these results may reflect an age-related maturation of myocardial tissue.

### Aging and epicardial fat accumulation

The first major novel finding from the current study was the increased EAT across the age-groups. Echocardiography-derived EAT is consistently and strongly associated with myocardial lipid content [15,43]. Previous autopsy findings have demonstrated that increasing levels of myocardial lipids is part of the normal aging process [44]. As lipid infiltration into the myocardium increases, LV myocardial function (such as longitudinal strain) is impaired [14,34]. In the present study, we found that increased thickness of EAT was associated with declining systolic and diastolic indices of LV myocardial mechanics. While the exact mechanisms through which EAT impacts myocardial mechanics are still speculative, early findings suggest that lipid accumulation in the epicardium causes enlargement of cardiomyocytes and thus impaired oxygen delivery. In turn, the lipid accumulation and hypoxia trigger several potential responses including: pro-inflammatory and pro-atherogenic cytokines in the myocardium, cardiomyocyte excitation-contraction coupling abnormalities from altered calcium handling, and increased levels of oxidative stress from reactive oxygen species [45-47]. Thus, through a series of cellular pathways, these reactive oxygen species expedite interstitial and peri-vascular fibrosis in the aging heart [48], further impairing myocardial mechanics.

### Aging and left ventricular dyssynchrony

Elevated levels of fibrosis and lipid content in the more mature myocardium may also slow signal conduction [48], consequently leading to LV-dyssynchrony. The second major novel finding in the current study was that longitudinal and circumferential LV-dyssynchrony at the base increased across the age-groups, independent of the effects of cholesterol, glucose, and hsCRP. Meanwhile, circumferential LV-dyssynchrony at the apex remained unchanged. Interestingly, we found age-related increases in apical circumferential strain and rotation, but no change in basal circumferential strain or rotation. This finding agrees with previous observations of the aging-heart [10]. Thus, the potential preservation of LV pump function may be predominantly governed by apical LV function, which is more resilient to LV-dyssynchrony, and augments deformation and rotation; leading to the aforementioned compensatory increase in LV twist.

### Associations between LV-dyssynchrony, myocardial fat, and myocardial mechanics

LV-dyssynchrony and EAT may be underlying factors responsible for altered myocardial mechanics in older healthy males. Only two MRI studies have observed increasing myocardial dyssynchrony in healthy aging people [17,18], while no studies have previously examined age-related changes in EAT in healthy males. We found the combination of EAT, longitudinal, and circumferential LV-dyssynchrony at the base explained more than one-third of the variability in longitudinal strain and LV twist, respectively. LV-dyssynchrony and EAT may therefore be valuable early indicators of LV dysfunction in healthy older people.

### Clinical significance

The potential effect of age on dyssynchrony may permit clinicians to accept a certain ‘normal’ level of LV-dyssynchrony in healthy older people. In the present study, half of the healthy older participants reached clinical threshold values for both longitudinal and circumferential STE dyssynchrony [25,26], despite our confirmation of preserved global systolic function across the age-groups [37]. Further research may be necessary to identify age-sensitive threshold values for LV-dyssynchrony.

### Study limitations

The cross-sectional design has limitations. While the number of participants in our study may appear limited, our sample size was carefully estimated and reached significance between groups. The discarding of some poor quality cine-loops, inherent to echocardiography, was a limitation; with a standard percentage of rejection [23], despite infrequent reporting in the literature [8,28]. A lack of echogenicity (clarity of acoustic ultrasound windows) in older participants precluded the capacity to confidently assess STE parameters in the radial axis and apical two-chamber and three-chamber windows. Intra and inter-observer variability was slightly high for twisting mechanics and LV-dyssynchrony, but not dissimilar to other studies reporting these variables [49]. However, the accuracy of assessing LV-dyssynchrony was increased by the use of several techniques (TDI, STE). Biological assessments were delimited to the most clinically important parameters. Thus, understanding the aging-heart may warrant further more comprehensive investigations, including larger scale longitudinal assessment of individuals as they age.

## Conclusion

We established a broad cardiac profile in healthy males of three different age-categories: demonstrating a likely age-associated decline in diastolic function and longitudinal deformation, with a compensatory increase in apical circumferential deformation, rotation and LV twist. For the first time with echocardiography, we showed increasing EAT, longitudinal, and circumferential-basal LV-dyssynchrony across three incremental age-groups of healthy males, independent of the effects of blood biology. We found these variables together significantly explained the variability in longitudinal deformation and LV twist (two major markers of LV mechanics), possibly through pro-inflammatory and pro-fibrotic pathways in the myocardium. Understanding the healthy aging-heart and its typical evolution may assist in the early distinction and prevention of cardiovascular diseases in healthy aging people.

## Competing interests

The authors declare that they have no competing interests.

## Authors’ contributions

TM, GN, and EC recruited participants. EC conducted all echocardiographic and clinical measurements. GN, FD and EC conducted statistical analyses. FD and EC drafted the paper, and GN and PO reviewed, corrected and helped finalise the manuscript. All authors read and approved the final manuscript.

## Pre-publication history

The pre-publication history for this paper can be accessed here:

http://www.biomedcentral.com/1471-2261/14/95/prepub
